# Association Between Adherence to Cancer Screening and Knowledge of Screening Guidelines: Feasibility Study Linking Self-Reported Survey Data With Medical Records

**DOI:** 10.2196/10529

**Published:** 2018-11-01

**Authors:** Aisha K Lofters, Deanna Telner, Sumeet Kalia, Morgan Slater

**Affiliations:** 1 Centre for Urban Health Solutions Li Ka Shing Knowledge Institute St. Michael’s Hospital Toronto, ON Canada; 2 Department of Family and Community Medicine St. Michael's Hospital Toronto, ON Canada; 3 Department of Family & Community Medicine University of Toronto Toronto, ON Canada; 4 Institute for Clinical Evaluative Sciences Toronto, ON Canada; 5 Dalla Lana School of Public Health Toronto, ON Canada; 6 South East Toronto Family Health Team Toronto, ON Canada; 7 University of Toronto Practice-Based Research Network Toronto, ON Canada

**Keywords:** cancer screening, electronic medical records, electronic survey, health literacy, self-reported data

## Abstract

**Background:**

It is possible that patients who are more aware of cancer screening guidelines may be more likely to adhere to them.

**Objective:**

The aim of this study was to determine whether screening knowledge was associated with the documented screening participation. We also assessed the feasibility and acceptability of linking electronic survey data with clinical data in the primary care setting.

**Methods:**

We conducted an electronic survey at 2 sites in Toronto, Canada. At one site, eligible patients were approached in the waiting room to complete the survey; at the second site, eligible patients were sent an email inviting them to participate. All participants were asked to consent to the linkage of their survey results with their electronic medical record.

**Results:**

Overall, 1683 participants responded to the survey—247 responded in the waiting room (response rate, 247/366, 67.5%), whereas 1436 responded through email (response rate, 1436/5779, 24.8%). More than 80% (199/247 and 1245/1436) of participants consented to linking their survey data to their medical record. Knowledge of cancer screening guidelines was generally low. Although the majority of participants were able to identify the recommended tests for breast and cervical screening, very few participants correctly identified the recommended age and frequency of screening, with a maximum of 22% (21/95) of screen-eligible women correctly answering all 3 questions for breast cancer screening. However, this low level of knowledge among patients was not significantly associated with screening uptake, particularly after adjustment for sociodemographic characteristics.

**Conclusions:**

Although knowledge of screening guidelines was low among patients in our study, this was not associated with screening participation. Participants were willing to link self-reported data with their medical record data, which has substantial implications for future research.

## Introduction

According to the Canadian Cancer Society, 23 Canadians are diagnosed with cancer each hour, resulting in 202,400 new cases of cancer diagnosed in 2016 alone [1]. Screening for cancer is an important tool in our efforts to prevent cancer mortality. A number of initiatives to increase screening uptake have been undertaken in the past few decades, including the introduction of centralized, organized screening programs for cervical (through the Pap test), breast (mammogram), and colorectal cancer (fecal occult blood testing, FOBT) in Ontario, Canada’s most populous province [2-4]. The provincial agency responsible for cancer services, Cancer Care Ontario, currently recommends that FOBT should be used for colorectal cancer screening, although those who have had a colonoscopy in the preceding 10 years are considered up-to-date [5].

Despite the known benefits of screening and the existence of organized screening programs in Ontario, the screening uptake in the province is still suboptimal. With the screening uptake currently estimated at 65% for breast cancer, 63% for cervical cancer, and 60% for colorectal cancer [6], more investigation is needed into how to improve screening rates. Various factors may impact the likelihood of screening. Health literacy has been shown to be associated with being within recommended cancer screening guidelines [7,8], and studies have shown an association between knowledge about cancer screening and screening uptake [9-13]. It is possible that patients who are more educated about screening guidelines may be more likely to adhere to them. For example, Hansen et al found that women who received cervical cancer screening were more likely to be aware of the recommended screening interval [14]. However, previous studies suggested that knowledge of screening guidelines is low in primary care [15-17]. Considering that primary care physicians play a central role in screening— performing Pap tests, distributing FOBT kits, and referring patients for mammography, as well as educating patients about screening and screening guidelines—it is important to understand if patients’ knowledge of cancer screening guidelines is associated with screening uptake. As self-report of cancer screening can often be inaccurate [18-21], we wanted to directly link self-reported survey responses to patients’ electronic medical record (EMR). However, attempting to link self-reported data to electronic clinical data is relatively new in the Canadian primary care setting. We were unsure as to how feasible this process was as well as how acceptable this would be to patients. As such, the specific objectives of this feasibility study were to assess the feasibility and acceptability of linking electronic survey data with clinical data in the primary care setting and determine whether cancer screening knowledge is associated with the documented screening participation among eligible participants.

## Methods

### Study Design

An electronic survey was developed to assess patients’ knowledge of cancer screening guidelines (see details below) at 2 primary care organizations in Toronto, Canada’s most populous city. At Site A, all patients presenting for appointments were approached to complete the survey on a tablet in the waiting room; at Site B, all eligible patients were sent an email inviting them to participate in the survey. Patients were also asked to consent to the linkage of their survey results with their EMR.

### Study Setting

This study was based at 2 distinct primary care organizations in the city of Toronto. Site A is a multidisciplinary multisite primary care practice that provides care to >35,000 patients in downtown Toronto. The practice serves many patients who are low income, homeless or underhoused, and living with addictions. Site B is a multidisciplinary primary care practice that provides care to approximately 19,000 patients in the eastern portion of Toronto. The practice serves a multicultural patient population that is mostly low-to-middle income. The practice has been collecting email addresses for patients over the age of 18 years since 2011, and at the time of the study, had email addresses for approximately 50% of their patient population. When patients provide their email, they are given an information letter and asked to sign a consent form, acknowledging the risks of email communication and conditions for appropriate use.

We used 2 sites to increase the generalizability of our findings through a larger and more diverse sample. In addition, these 2 sites allowed us to evaluate 2 different methods of patient recruitment (tablet vs email), especially as the use of email contact for research purposes is still relatively new; however, comparing results between sites was not a study objective.

### Eligibility Criteria

Patients of the primary care practices were eligible to participate in this study if they were eligible for cancer screening based on their age and sex (women aged 21-74 years and men aged 50-74 years). An additional criterion for Site B was that they had to have a documented email address in their medical record. The eligibility criteria based on age and gender match provincial screening guidelines; women are eligible for cervical cancer screening if they are aged 21-69 years and for breast cancer screening if they are aged 50-69 years. Men and women are eligible for colorectal cancer screening if they are aged 50-74 years [2,22,23].

This survey was offered to all eligible patients presenting for appointments at Site A over a 5-month period (March-August 2016) and was emailed to all eligible patients with email addresses on file at Site B on September 19, 2016. The email survey was open until October 20, 2016.

### Survey

The survey was adapted from a previous survey used in the preliminary work conducted at Site A [15]. It included questions regarding knowledge of the 3 evidence-supported guidelines of cancer screening in Ontario [15], and sociodemographic questions including immigration status, ethnicity, and financial strain [24]. Specifically, knowledge of guidelines was assessed by asking about the age of the screening eligibility, screening modality, and frequency of screening for each of cervical, breast, and colorectal cancer (see Textboxes 1-3). At the end of the survey, participants were asked if they consented to have their survey responses linked to their medical chart to assess their screening history. Once participants completed the survey, they were given information outlining the current screening guidelines in Ontario.

All survey data were collected utilizing the Ocean Studies platform, a cloud-based software program [25], which is integrated with the EMR. The platform allows self-reported patient data to be collected through the use of a secure and unique identifier through a “pseudonymization” process, which allows each patient to be identified without having to store any information that could identify the patient outside of the EMR. At Site A, eligible patients were identified from the EMR and approached to complete the survey on a tablet at the time of checking in to the front desk. At Site B, eligible patients were sent an email through the Ocean Studies platform, which contained a link to the survey. It was not possible for patients to reply to the email.

### Chart Review

For the subset of patients consented, we extracted data on their cancer screening history, including the date of the most recent cancer screening, from their electronic chart using the automated search feature in the EMR.

### Data Analysis

Responses to each of the screening knowledge questions were categorized as being correct or incorrect, and a count of the number of correct questions for each cancer screening type among those eligible for that type of screening was calculated as a measure of the screening guideline knowledge. Descriptive analyses were performed to describe the demographics and characteristics of the study participants, including their knowledge of current screening guidelines.

**Questions on screening eligibility age, screening modality, and frequency of screening for breast cancer. The bolded text represents the correct response**

The following questions are about current breast cancer screening guidelines:

1. The screening test for breast cancer is:
  - Ultrasound
  - Magnetic resonance imaging (MRI)
  - **Mammogram**
  - Breast exam by doctor/nurse
  - Unsure/don’t know
2. Between what ages should women of average risk be screened for breast cancer:
  - 20-74 years
  - 30-74 years
  - 40-74 years
  - **50-74 years**
  - Unsure/don’t know
3. How often should women have a screening test for breast cancer?
  - Every 6 months
  - Every 1 year
  - **Every 2 years**
  - Every 3 years
  - Unsure/don’t know

**Questions on screening eligibility age, screening modality, and frequency of screening for cervical cancer. The bolded text represents the correct response.**

The following questions are about current cervical cancer screening guidelines:

1. The screening test for cervical cancer is:
  - Fecal occult blood test
  - **Pap test**
  - Pelvic ultrasound
  - Pelvic exam
  - Unsure/don’t know
2. Which of the following women should be screened for cervical cancer?
  - Those who are at least 18 years old and who have ever been sexually active
  - **Those who are at least 21 years old and who have ever been sexually active**
  - Those who are at least 21 years old, whether or not they have been sexually active
  - Those who are at least 25 years old, whether or not they have been sexually active
  - Unsure/don’t know
3. How often should women have a screening test for cervical cancer?
  - Every 6 months
  - Every 1 year
  - Every 2 years
  - **Every 3 years**
  - Don’t know

To assess the association between screening knowledge and documented screening behavior, we conducted a subgroup analysis of participants who consented to the linkage to their EMR. The dates of participants’ last screening test(s) were used to categorize participants as being up-to-date on screening (ie, a Pap test in the previous 3 years, a mammogram in the previous 2 years, or an FOBT in the previous 2 years) [26]. We assessed the association between screening uptake and screening knowledge (the count of correct answers) for patients who were eligible for each type of screening using the Cochrane-Armitage trend test [27]. Multivariate regression analyses (logistic or log-binomial, as appropriate) were used to assess associations between screening uptake and knowledge, adjusting for age, income, immigration status, and ethnicity. We decided *a priori* to adjust for these variables as they have been shown in the literature to be significantly associated with cancer screening uptake [28-33]. Logistic regression is not the most suitable statistical analysis when the outcome is common (as it was for breast cancer screening), as it can contribute to the underestimation or overestimation of the true effect [34,35]. Hence, a log-binomial regression model was fitted for breast cancer screening.

All statistical analyses were conducted using SAS version 9.4 (SAS Institute Inc, Cary, NC, USA) and *P*<.05 was considered statistically significant. Research policies at the practice sites where the research was conducted require that any individual cells in a table with a numerical value of ≤5 cannot be reported, to reduce the risk of identifying participants. As such, all cell sizes <5 were suppressed.

### Ethics

This study was approved by the Research Ethics Board at St. Michael’s Hospital and Michael Garron Hospital, which is associated with the South East Toronto Family Health Team.

**Questions on screening eligibility age, screening modality, and frequency of screening for colorectal cancer. The bolded text represents the correct response.**

The following questions are about current colorectal cancer screening guidelines:

1. The recommended screening test for adults of average risk of colorectal cancer is:
  - **Fecal occult blood test**
  - Rectal exam
  - Abdominal ultrasound
  - Colonoscopy
  - Unsure/don’t know
2. When should adults of average risk start being screened for colorectal cancer?
  - 40 years of age
  - 45 years of age
  - **50 years of age**
  - 55 years of age
  - Unsure/don’t know
3. How often should adults be screened for colorectal cancer?
  - Every 1 year
  - **Every 2 years**
  - Every 3 years
  - Every 10 years
  - Don’t know

## Results

**Figure 1:**
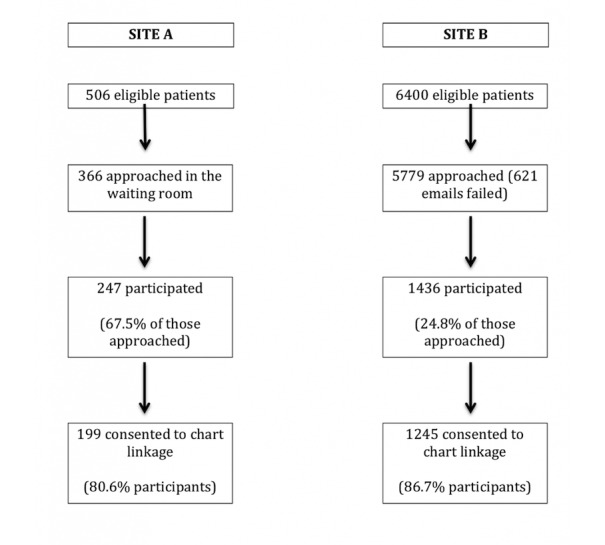
Study recruitment.

In total, 506 eligible patients were seen in waiting rooms at Site A during the recruitment period and 6400 eligible patients with email addresses identified at Site B (Figure 1). The response rate significantly differed between the 2 sites—67.5% (247/366) of those approached in the waiting room versus only 24.85% (1436/5779) of those approached by email participated. However, the absolute number of study participants was much lower through recruitment in clinic (247 participants at Site A) than through the use of email (1436 participants at Site B). More than 80% (199/247, 80.6%, at Site A and 1245/1436, 86.70%, at Site B) of participants at both sites were willing to link their survey responses to their medical chart.

Table 1 describes the demographic characteristics of study participants overall and at the 2 study sites. Female participants were predominant at both sites, in line with 2 of the 3 evidence-based cancer screening actions being targeted at females only, and roughly 20% (328/1683) of participants at both sites reported having not enough income at the end of the month. More than 20% (374/1683) of participants were foreign-born at both sites, but participants at Site A were twice as likely to identify as visible minorities (58/247, 23.5%, vs 172/1436, 11.98%).

**Figure 2:**
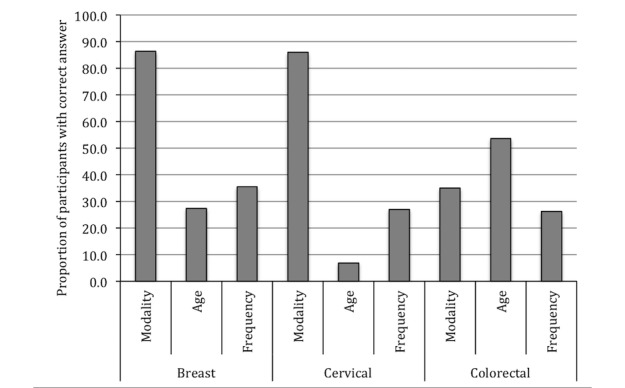
Proportion of participants who correctly identified the test modality, age and frequency of breast, cervical and colorectal cancer screening guidelines.

Knowledge of cancer screening guidelines was generally low. Respondents were likely to be able to report the recommended tests for breast (1453/1683, 86.33%) and cervical (1447/1683, 85.98%) cancer screening (Figure 2). However, very few participants correctly identified the age and criteria at which cervical cancer screening should begin (116/1683, 6.89%). Only 35.06% (590/1683) participants were able to correctly identify FOBT as the recommended test for colorectal cancer screening, with 49.32% (830/1683) naming colonoscopy as the appropriate screening test for colorectal cancer. The proportion of patients correctly responding to questions was consistently lower at Site A than at Site B; for example, 76.1% (188/247) patients at Site A identified mammogram as the recommended test for breast cancer screening versus 88.09% (1265/1436) of patients at Site B.

**Figure 3:**
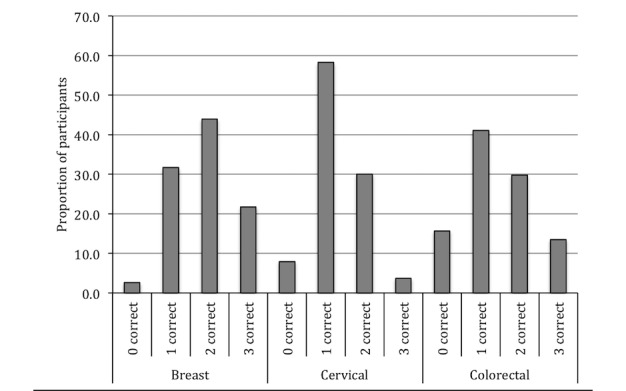
Percentage of study participants by the number of questions answered correctly stratified by screening type.

When we considered an overall measure of screening knowledge (the count of correct responses), more participants answered zero questions correctly than answered all questions correctly aside from those women eligible for breast cancer screening (Figure 3). Figure 3 shows percentages of study participants among 464 participants eligible for breast cancer screening, 1344 for cervical cancer screening, and 770 for colorectal cancer screening. Participants were most likely to get zero questions correct for colorectal cancer screening. While screen-eligible women were most knowledgeable about breast cancer screening, only 22% (21/95) answered all 3 questions correctly for breast cancer screening. However, the majority of participants knew, at least, one fact about each screening type they were eligible for. Knowledge levels appeared to be different between the 2 sites, with a higher proportion of respondents at site A being unable to answer any questions correctly for all 3 cancer types.

**Figure 4:**
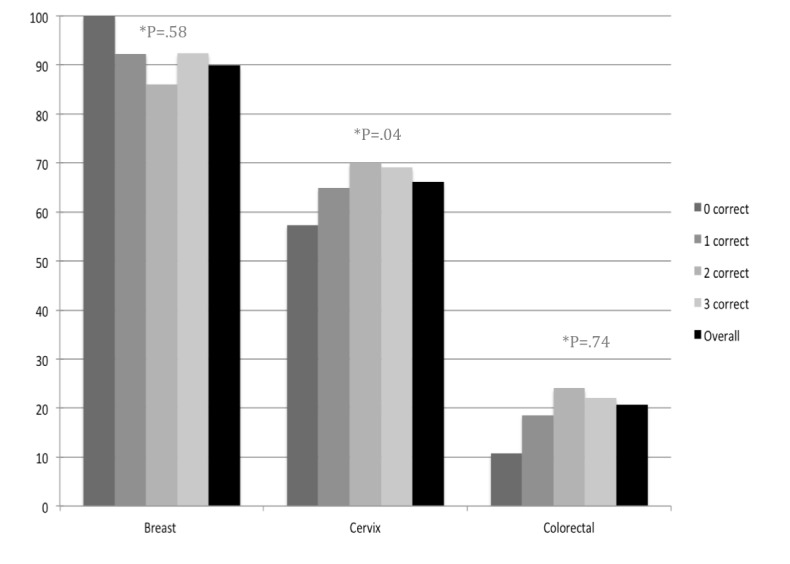
Percentage of study participants at Site B who were up-to-date on cancer screening by modality and the number of questions correctly answered relative to each modality.

While over 80% (199/247, 80.6%, at Site A and 1245/1436, 86.70%, at Site B) of participants agreed to the linkage of their survey responses to their clinical data, technical issues at Site A did not allow for the actual linkage to occur (EMR identification numbers were not retained because of software malfunction). As such, the analysis of the association between screening knowledge and uptake is limited to 1245 participants at Site B who consented to the chart linkage. Among these patients, the screening uptake among screen-eligible participants ranged as follows: 20.7% (119/576) for colorectal, 66.21% (672/1015) for cervical, and 89.9% (319/355) for breast cancer screening. The level of knowledge of screening guidelines appeared to have no association with breast cancer or colorectal cancer screening (Figure 4). However, increasing knowledge was associated with an increase in the cervical screening uptake (*P*=.04).

When adjusting for age, income, immigration status, and ethnicity, the number of questions answered correctly was not significantly associated with the screening uptake for any cancer screening type (Table 2). In addition, age, income, immigration status, and ethnicity were not statistically significant in the models, except for age for cervical cancer screening, where women aged 30-39 years were more likely to be up-to-date than women aged 60-69 years (adjusted odds ratio 3.24, 95% CI 1.95-5.39).

**Table 1:** Demographic characteristics of study participants, overall and broken down by the site.

Characteristics		Overall^a^ (n=1683)	Site A (n=247)	Site B (n=1436)
**Gender, n (%)**				
	Male	251 (14.91)	47 (19.03)	204 (14.21)
	Female	1393 (82.77)	183 (74.09)	1210 (84.26)
**Age in years, n (%)**				
	21-29	142 (8.37)	27 (10.93)	115 (8.01)
	30-39	409 (24.30)	51 (20.64)	358 (24.93)
	40-49	336 (19.96)	37 (14.98)	299 (20.82)
	50-59	388 (23.05)	63 (25.51)	325 (22.63)
	60-69	302 (17.94)	40 (16.19)	262 (18.25)
	>70	81 (4.81)	14 (5.67)	67 (4.67)
**Immigration status, n (%)**				
	Foreign born	374 (22.22)	64 (25.91)	310 (21.59)
	Canadian born	1279 (75.99)	164 (66.40)	1115 (77.65)
**Race, n (%)**				
	Caucasian	1359 (80.74)	157 (63.56)	1202 (83.70)
	Other	230 (13.67)	58 (23.48)	172 (11.98)
**Income at the end of the month, n (%)**				
	More than enough	530 (31.49)	68 (27.53)	462 (32.17)
	Just enough	579 (34.40)	69 (27.94)	510 (35.52)
	Not enough	328 (19.49)	51 (20.65)	277 (19.29)

^a^Not all questions were answered by all participants, and proportions will not add up to 100%.

**Table 2:** Results of multivariate regression analyses for the 3 types of cancer screening among study participants at Site B who agreed to the chart linkage. Other variables in the model included age, income, immigration status, and ethnicity.

Correct responses	Breast adjusted relative risks (95% CI)	Cervical adjusted odds ratios (95% CIs)	Colorectal adjusted odds ratios (95% CIs)
0	N/A^a^	0.82 (0.29-2.28)	0.87 (0.31-2.40)
1	0.99 (0.90-1.10)	0.82 (0.37-1.84)	1.46 (0.68-3.14)
2	0.93 (0.84-1.02)	0.87 (0.38-1.99)	1.62 (0.74-3.55)
3	1.0	1.0	1.0

^a^N/A: not applicable; of women who consented to the chart linkage and who had complete data for all covariates, only 8 women had zero questions correct.

## Discussion

This study found that knowledge of cancer screening guidelines, specifically appropriate screening ages, modalities, and time intervals, was low among primary care patients. Very few patients correctly identified the recommended test, age and frequency of screening for breast, cervical, or colorectal cancer, with a maximum of 22% (21/95) of screen-eligible women correctly answering all 3 questions for breast cancer screening. Knowledge was particularly low for colorectal cancer screening, where more patients selected colonoscopy as the appropriate screening test compared with FOBT. However, this low level of knowledge among patients was not significantly associated with screening uptake, particularly after adjustment for sociodemographic characteristics.

Although educating patients about the benefits of screening has been shown to improve screening uptake [10,36-39], our findings suggest educating patients on the specific details of screening guidelines may not be a meaningful way of increasing adherence. Patient education interventions that have been shown to be effective for increasing screening have conveyed information on the epidemiology of relevant cancer, indications for screening including guidelines, details of the screening test, risks and benefits of screening, and ways to overcome potential barriers to screening [10,38,39]. Knowledge of these factors may directly motivate eligible adults to pursue screening [39] and thus is likely more important in improving adherence than knowing the appropriate age, test, and time interval. This is particularly relevant in the primary care setting where the provider can alert screen-eligible patients to when they are next due and order the appropriate test. Furthermore, provider recommendation for screening has been shown to improve screening [40].More than 80% (199/247, 80.6%, at Site A and 1245/1436, 86.70%, at Site B) of patients were willing to share their EMR data with the research team and link their survey responses to their personal health information. As EMRs become pervasive, these numbers hold promise for future research studies in the primary care setting making use of these data. The use of anonymized EMR data on its own without the need for patient consent is common [41,42], but this study is an example of how identifiable records can be combined with self-reported data to produce useful findings.

We used 2 different methods to reach patients in this study—recruiting patients in the waiting room and by email. Although our response rate was much higher when offering the survey to patients in the waiting room than by email (247/366, 67.5%, vs 1436/5779, 24.85%), which was expected [43,44], we were able to reach many more patients with the email survey and thus have much higher absolute numbers. An email survey allows participants to complete it at their own pace, as opposed to in the waiting room, where surveys may be abandoned if patients are called in to be seen by their provider. Sending the survey by email also required much less staff time. Several research staff recruited patients at Site A over the course of several months, whereas all emails at Site B were sent to all eligible patients in a single batch. These results suggest that using email is a feasible way of recruiting patients for clinical studies and more time-efficient and cost-efficient than recruitment in the waiting room. Moreover, email surveys have the added advantage of allowing for recruitment of patients who come into the office infrequently and would be unlikely to be captured in waiting room surveys. As EMRs become more advanced, it is quite possible that in the future, practice-based researchers will be able to identify patients with particular medical diagnoses through EMR searches and email them regarding participating in relevant studies.

In health care, electronic communication is still relatively new, and most research has focused on the acceptability of utilizing email for clinical purposes as opposed to research [45,46]. The use of email to communicate with patients for research purposes still presents challenges. It is not possible to guarantee the privacy and security of email messages and know whether the email will be received by the one it is intended. Although our survey contained no personal health information, it did identify each participant as a patient of the primary care site. Not surprisingly, the Canadian Medical Practice Association has highlighted email communication with patients as holding potential legal risks, and the privacy commissioner of the province of Ontario has indicated that email communication with patients is not recommended, but if to be used, requires appropriate safeguards and security procedures to be in place [47]. Other practical issues include ensuring that email addresses are kept up-to-date, assessing if patients with email addresses are representative of the practice as a whole, determining an appropriate age of consent for email communication, updating email addresses as this age of consent is reached for pediatric patients and monitoring the total number of emails being sent out to patients by the practice, whether for research or other purposes, to avoid email fatigue.

This study has several limitations. First, we were unable to link surveys at one site to patient charts because of technical issues with the software. Despite significant advances in technology in recent years, unfortunately software glitches and malfunctions are still common. Second, the survey was available in only English, so patients who were not able to read English well were unlikely to participate. Third, we did not consider colonoscopy as a correct answer to the recommended screening test or as a measure of being up-to-date on colorectal cancer screening because it is not recommended by the provincial cancer agency for patients at average risk. However, almost half of the patients thought this was the recommended test and patients with colonoscopy in the past 10 years do not require FOBT screening. Anecdotally, many primary care providers at the participating site use colonoscopy for screening, which likely explains the very low FOBT screening rate (119/576, 20.7%) that we observed.

Although knowledge regarding the age of initiation, recommended screening test, and time interval for breast, cervical, and colorectal cancer screening was low among primary care patients in this study, this was not associated with a lack of screening participation. It is possible that physicians’ recommendation and meeting a minimum threshold of screening knowledge may be sufficient to facilitate screening uptake. For example, if a 40-year-old woman knows that she should have a Pap test at least once every 3 years, it may not matter if she believes screening should start at the age of 18 years instead of 21 years. We also found that patients were willing to link self-reported data with their medical record data, which has significant implications for the possibilities for future research. Future research that builds directly on the findings from this study should use the EMR to identify patients in primary care practices who meet screening criteria and are due for screening, send them email reminders with accompanying evidence-based educational information on screening, and assess the effect on screening uptake.

## Abbreviations

EMR: electronic medical record
FOBT: fecal occult blood testing
